# Targeted fetal cell‐free DNA screening for aneuploidies in 4,594 pregnancies: Single center study

**DOI:** 10.1002/mgg3.678

**Published:** 2019-05-08

**Authors:** Altug Koc, Ozge Ozer Kaya, Berk Ozyilmaz, Yasar B. Kutbay, Ozgur Kirbiyik, Taha R. Ozdemir, Kadri M. Erdogan, Merve Saka Guvenc, Deniz C. Oztekin, Mehmet Ozeren, Halil G. Pala, Atalay Ekin, Cenk Gezer, Alkim G. Sahingoz Yildirim, Bahar Konuralp Atakul, Secil Kurtulmus, Ugur Turhan, Cuneyt E. Taner

**Affiliations:** ^1^ Genetic Diagnosis Center Tepecik Training and Research Hospital Health Sciences University Izmir Turkey; ^2^ Perinatology Clinic Department of Obstetrics and Gynaecology Tepecik Training and Research Hospital Health Sciences University Izmir Turkey

**Keywords:** cfDNA screening, NIPS, NIPT

## Abstract

**Background:**

Next‐generation sequencing (NGS) and discovery of fetal cell‐free DNA (cfDNA) in the maternal circulation render possible prenatal screening for trisomy 21 (Down syndrome), trisomy 18, trisomy 13, and sex chromosome aneuploidies. The approach is called “fetal cfDNA screening” and in contrast to noninvasive conventional serum screening, it provides the identification of 98%–99% of fetuses with Down syndrome.

**Methods:**

Retrospective analysis of targeted noninvasive prenatal testing (NIPT) (Clarigo Test) pregnancies with moderate risk, which we have reported between 2016 and 2018 years is presented. Two separate laboratory workflows and NGS platforms are used for the same targeted NIPT analysis.

**Results:**

In total, 4,594 pregnant women were investigated. Initial 3,594 cases are studied by MiSeq platform, the last 1,000 cases by NextSeq. Failure rate for MiSeq platform is 10.9% and for NextSeq is 8.7%. Automatically reported cases constitute 75% of the MiSeq group and 87% of the NextSeq group.

**Conclusions:**

Targeted NIPT results suggest that MiSeq platform could be used for NIPT which would be an essential option particularly for laboratories with low sample flow. And, the NextSeq platform has easier wet lab process and also increased success rate in automatic reporting which is suitable for centers with high number of NIPT cases.

## 1 INTRODUCTION

About one of every 150 live births has chromosomal abnormalities and the most common disorder is Down syndrome (trisomy 21). Its incidence increases by maternal age (Carlson & Vora, 2017). Unfortunately, our prenatal efforts to prevent aneuploidies just keep the live birth rates stable over time (Loane et al., 2013). The history of prenatal genetic testing includes first invasive prenatal karyotyping in 1967 (Jacobson & Barter, 1967). Although the invasive tests precisely determined the disorders of fetal chromosomes, risk assessment and patient classification by a noninvasive screening strategy were needed before invasive procedures due to their complication risks. Main exception was the presence of major fetal anomaly; in that case, invasive test with microarray had higher diagnostic yield (Salomon et al., 2017). Keeping invasive diagnostic tests in second line in most of the pregnancies, resulted in low‐detection rates of aneuploidies due to low‐detection rates of conventional maternal serum screening and fetal ultrasonography. So, there was a great need for a noninvasive screening with higher detection capability. The solution was the discovery of placenta‐derived fetal DNA in maternal plasma (Lo et al., 1997) and fetal aneuploidy detection by next‐generation sequencing (NGS) is achieved in 2008 (Chiu et al., 2008; Fan, Blumenfeld, Chitkara, Hudgins, & Quake, 2008). Commercial noninvasive prenatal testing (NIPT) products became publicly available by the year 2011 (Carlson & Vora, 2017).

NGS based, cell‐free fetal DNA screening methods use two major strategies: First one is the genome‐wide (shotgun) random sequencing and tag‐counting approach. And the second one is targeted sequencing, which may be performed by “multiplex PCR targeting single‐nucleotide polymorphisms (SNPs)” or “selection of targeted regions via locus‐specific oligonucleotides.” All of the methods include substantial bioinformatics data processing pipeline which counts polymorphic genetic signatures and digitally segregates maternal and fetal cell‐free DNA (cfDNA) (Vermeesch, Voet, & Devriendt, 2016). Relative quantification of alleles identifies aneuploidies. Digital segregation is mandatory because there is no widely used physical separation technique for maternal and fetal cfDNA; however, if we achieve efficient, physical extraction of fetal cfDNA then a new era will begin.

In genome‐wide sequencing, high number of investigated polymorphic genetic loci (millions) in contrast to targeted sequencing (thousands) gives the opportunity of testing the whole chromosomes not just the common trisomies. In fact, by increasing the loci and subsequently the cost, cfDNA screening does not turn into a diagnostic test. The reason is not the technology, but the biology. The biologic causes of the false results include inconsistent fetal and placental genotype, medical conditions of the mother which elevate the maternal part of the cfDNA in plasma or decrease the rate of fetal fraction: Autoimmune diseases, cancers, and obesity. In addition, incidental maternal copy number variations (CNV), true fetal mosaicism, twin pregnancies, and transplantation may also affect the results (Bianchi & Chiu, 2018). For the sake of not missing fetal trisomies, the NIPT is designed to be highly sensitive and the gain of the sensitive design is low false‐negative rates (12%), but the cost of sensitive approach is the high false positives (88%) (Hartwig, Ambye, Sørensen, & Jørgensen, 2017). In the common practice, an invasive confirmatory test follows a positive NIPT result and if it shows a false positive then uniparental disomy (UPD) investigation of the involved chromosome is needed. If the cfDNA result is negative, further invasive investigation is not needed. Uncertain results deserve additional prenatal tests including invasive ones. Generally, the test may be offered to pregnant from the 10th week of gestation until delivery. Pre‐ and posttest genetic counseling should be given.

Although, there is a great opportunity to test fetal microdeletions and microduplications by NIPT, it is not recommended for routine use, clinic validation studies are needed (Gregg et al., 2016). Fetal cfDNA may also be used for the diagnosis of monogenic disorders. Paternally inherited or de novo mutant sequences are targeted in this method and the proof of concept studies is present in literature (Drury et al., 2016).

To date, approximately 4–6 million pregnants have got fetal cfDNA screening (Green, Rubin, & Olson, 2017). And, recent studies reveal there is 40%–70% decrease in invasive prenatal procedures since 2012 (Hui, Hutchinson, Poulton, & Halliday, 2017; Warsof, Larion, & Abuhamad, 2015). Though its efficiency, cfDNA screening is still so expensive in contrast to conventional maternal serum screening and the cost is the major obstacle to be used as a first‐tier screening test. Public finance strategies differ among governments (Chitty et al., 2016; Neyt, Hulstaert, & Gyselaers, 2014; Oepkes et al., 2016). Birth rates of different countries seem to be a decisive factor for public fund. For cost‐effective usage of NIPT in routine practice, distinct approaches are offered. Contingent model which combines first trimester screening with cfDNA screening is promising but there is no worldwide consensus yet (Huang, Meschino, Teitelbaum, Dougan, & Okun, 2017).

The birth rates of our country are the leading one in Europe, which has 1.4 million births per year (2015 data). And the first cfDNA screening practice is occupied in Genetic Diagnosis Center of Izmir Tepecik Training and Research Hospital in Aegean region.

## 2 MATERIALS AND METHODS

### 2.1 Patients' selection

The study approved by “Health Sciences University, Izmir Tepecik Training and Research Hospital Local Ethical Committee” and the participants gave informed consent. The study includes the retrospective investigation of 4, 594 pregnant women who have been followed by perinatology and medical genetics clinics and have fetal cfDNA screening test between January 2016 and March 2018. There is no nationwide accepted guideline for NIPT, so the indications for noninvasive prenatal screening test are determined by perinatology clinic and “Tepecik Criteria” includes: Advanced maternal age (≥35 years); having combined trisomy 21 risk between 1/300 and 1/1,000 or sole biochemical risk more than 1/1,000 with a normal fetal nuchal translucency (NT) in first trimester screening; having one fetal soft marker on ultrasonography for trisomy 21. The exclusion criteria are as follows: Major fetal anomaly or having fetal NT ≥ 3.5 mm; multiple pregnancy; vanishing twin; pregnancy by oocyte donation; ≤8 weeks of gestation; body mass index ≥ 35; parental chromosome anomaly; history of blood transfusion, transplantation, stem‐cell therapy, immunotherapy, radiotherapy in last 3 months; maternal malignancy.

### 2.2 Pretest counseling of patients

All of the patients are initially assessed in perinatology clinic and cases with NIPT indications are referred to medical genetics. Pre‐ and posttest genetic consultations are given according to suggestions of “National Society of Genetic Counselors.” Samples of “Informed Consent,” “Test Order Form,” and “Report” documents in Turkish are shared as [Link], [Link], [Link].

### 2.3 NIPT for trisomy 13, 18, and 21

In total, 4,594 cases are tested. Majority of them (3,594) are studied by MiSeq NGS (Illumina) platform and the last 1,000 of the cases are investigated by NextSeq (Illumina). The features of cases and their indications for NIPT are summarized in Table 1. The NGS platforms differ in their capacity: Ten to 12 cases are studied per a MiSeq run and the number is 55–60 cases per a NextSeq run. During the setup period of the test, first 100 NIPT cases have also invasive prenatal test for the validation of laboratory workflow.

**Table 1:** Features of 4,594 cases and their indications for NIPT

Features	Mean value (range)
Maternal age	32.3 years (16–50)
Weeks of gestation	16.2 (9–32)
Pregestational maternal weight and BMI	67.2 (37–110) and 25.5 (14.9–39)
cfDNA concentration	0.47 μl/ng (0.1–1.4)
Fetal fraction	6.2% (0.1–20.5)
Sample coverage^a^	2,197,285 (1,500,000–4,000,000)
Fetal sex ratios	Female: 50.3% and male: 49.7%

Indications for NIPT	Ratios, %
MSSR^b^	44.6
AMA^c^	31.3
Sole minor anomaly in fetal ultrasonography	19.64
Maternal anxiety	1.53
Others (family history, IVF pregnancy, decision of perinatology council etc.)	2.93

*Notes*

NIPT: noninvasive prenatal testing; BMI: body mass index; cfDNA: cell‐free DNA; IVF: in vitro fertilization.

^a^The data represents the performance of MiSeq platform. It increases up to 2.5–3 M in NextSeq platform. ^b^MSSR: maternal serum screening result; the number includes the cases with both MSSR and AMA which is 552. ^c^AMA: advanced maternal age. Fetal sexes are not reported.

Venous blood samples of the pregnants are drawn just in the hospital. Cargo delivery is not used for sample collection. The blood samples are collected in Streck tubes till afternoon; in the same day 3–4 ml plasma isolations are achieved and plasma are stored in −80°C until cfDNA extraction. If the density of cfDNA is not appropriate (normal range 0.1–0.8 ng/μl), resampling is needed. The workflow is performed according to the recommendations of the producer (Clarigo^™^, CE‐IVD Marked, Multiplicom, Belgium) and it includes the investigation of 4,000 SNPs: 600 for each of the 21, 18, 13, X chromosomes and 1,600 for reference chromosomes. Laboratory workflow consists of multiplex and universal PCRs. The amplicons are about 65–85 bp in length and the PCR steps are followed by magnetic bead purification. Subsequently, pooling of amplicon libraries and massively parallel sequencing are done. Cloud based “Clarigo Reporter, the Initial Version (Multiplicom, Belgium)” software is used for data analysis. If there is an inconvenient cfDNA, resampling is done in all cases. If there is a failure in initial test, second trial of whole steps is done; the exceptions are the pregnants with advanced gestation (≥20 weeks); they are offered invasive testing immediately following a failed NIPT.

### 2.4 Methods of data analysis

The Clarigo Reporter software makes quality control (QC), determination of fetal fraction, trisomy calling for chromosome 21, 18, 13, and optional fetal gender calling. The genders of fetuses are not determined. The bioinformatic analysis depends on the correlation of samples which are studied in the same conditions. The deviation from the common outcome is used as the indicator of trisomy. So to get an automatically called result, correlation with other samples, having a fetal fraction ≥4% and at least 2 M reads per sample is needed. The cases with “fetal fraction ≥4%” and “*Z*‐score > 3.5 (4 for Chr 13) and the trisomy evidence is ≥0.5” are automatically called “Positive” for the related trisomy and the cases with “*Z*‐score < 3.5 (4 for Chr 13) and the trisomy evidence ≤−2” are called “Negative.” Negative predictive value and positive predictive value are calculated manually for each patient and reported. In other cases, “low fetal fraction (LFF)” and “not automatically called” results are obtained and manual reportings are needed in LFF. “Uncertain” result is called when there is no clear outcome for the tested case. Also these “Rejected Samples” are due to low sample correlation.

This study has been performed with the “Initial Version Clarigo Reporter Software,” in the meantime a new version, Clarigo v2, is launched. The “Clarigo v2” includes new features and has improvements: Increased calling rate, lower cut‐off for fetal fraction to 3%, and a new QC report to provide more insight information on the performance of the test.

### 2.5 Posttest counseling and methods used for conformation of NIPT results

The results of the tests are given by posttest counseling and routine perinatology follow‐up is offered for *negative cases*; invasive diagnosis is offered for *positive reports*. Failed test results are reported as “Uncertain” and the patients are counseled about their options, risks and invasive prenatal testing is offered. Invasive testing includes “Quantitative Fluorescent Polymerase Chain Reaction (QF‐PCR)” and karyotyping by amniocentesis for the majority of the cases. For the rare cordocentesis and chorion villus sampling cases, maternal contamination is always excluded by short tandem repeat (STR) markers. *STRs are also used to investigate UPD* *in false‐positive cases*. Paternal blood is needed for heterodisomic UPD investigation.

## 3 RESULTS

For the setup, first 100 cases with a prenatal invasive test results are investigated as a demo study. Five of them have fetal trisomy 21‐positive result, four of the cases are automatically detected but one of them needs “manual reporting” due to LFF which is 3.8%. All of the cases confirmed as positive by amniocentesis. And, all of the trisomic fetuses are detected by NIPT in the demo group. The cases with positive results in demo study are summarized in Table 2. First 3,594 cases are studied by MiSeq platform, the last 1,000 cases by NextSeq and the results are presented in Table 3. Failure rate for MiSeq platform is 10.9% and for NextSeq is 8.7%. Automatically reported cases constitute 75% of the MiSeq group and 87% of the NextSeq group.

**Table 2:** The features of initial five cases with positive results in demo study^a^

Case No.	Maternal age	Weeks of gestation	Indication of NIPT	Invasive test result
1	40	16	AMA	Trisomy 21
2	39	16	FTS^b^: 1/150 (NT^c^: 1.05 mm) and AMA	Trisomy 21
3	34	16	FTS: 1/50	Trisomy 21
4	18	16	FTS: 1/147	Trisomy 21
5	30	16	FTS: 1/50	Trisomy 21

*Notes*

AMA: advanced maternal age; NIPT: noninvasive prenatal testing.

^a^Demo study includes 100 cases with invasively confirmed results. All of the five trisomy 21 cases are detected. ^b^FTS: combined fetal trisomy 21 risk in first trimester maternal serum screening. ^c^NT: nuchal translucency at 12th week of gestation. Case No. 5 is reported manually due to low fetal fraction (3.8%), the others are automatically reported. All of the results are confirmed by amniocentesis.

**Table 3:** Results of 3,594 cases by MiSeq platform and 1,000 cases NextSeq platform

Test result^a^	MiSeq platform (3,594 cases)	NextSeq platform (1,000 cases)	Total (4,594 cases)
Normal (negative)	2,714 (75.51%)	873 (87.3%)	3,587 (78.08%)
False‐negative trisomy 21	1	0	1
False‐negative trisomy 18	1	0	1
Trisomy (positive)	37 (1.02%)	5 (0.5%)	42 (0.91%)
Trisomy 21	19	4	23
True	15	0	15
False	2	2	4
Unconfirmed	2	2	4
True trisomy 13	12	0	12
True	1	0	1
False	6	0	6
Unconfirmed	5	0	5
True trisomy 18	6	0	6
True	2	0	2
False	0	0	0
Unconfirmed	4	1	5
Uncertain result for trisomy 13	243 (6.76%)	20 (2%)	263 (5.72%)
Uncertain result for trisomy 18	135 (3.75%)	7 (0.7%)	142 (3.09%)
“Manual reporting” due to low fetal fraction (3%–4%) or low sample coverage (1.5–2 M reads) or uncertain for both trisomy 13 and 18	72 (2%)	8 (0.8%)	80 (1.74%)
Test failure	393 (10.9%)	87 (8.7%)	480 (10.44%)
Uncertain result for trisomy 21	127	12	139
Low fetal fraction (<3%)	90	23	113
Low cell‐free DNA concentration	73	18	91
Rejected due to “low sample correlation”	39	34	73
Low sample coverage (<1.5 M reads)	16	0	16
Other reasons	48	0	48

^a^Confirmation of “normal (negative) test results” is done by follow‐up of the neonate. More than 90% of the pregnant with “normal results” have their delivery in our hospital but there is no exact number for them. “Positive test results” are confirmed by invasive prenatal diagnosis except one case which has a neonate with Down syndrome phenotype. Some of the cases with positive results do not prefer invasive testing for confirmation and leave follow‐up; they are mentioned as “unconfirmed” in the table. There is no case with a positive result for more than one trisomy. Uncertain test results for trisomy 21 are regarded as test failure but trisomy 13 and 18 uncertain results are reported.

## 4 DISCUSSION

Our center has “first in house NIPT” in our country, so the setup process has its own obstacles. Cluster density optimization and MiSeq maintenance are the major technical issues. Because of the missing legal regulations regarding NIPT in our country, “Tepecik Criteria” is constituted by the permission of Health Ministry which is given under “Patients' Selection” heading. The indication group mainly includes “pregnants who have moderate risk for trisomy.” But, it should be extended to include high‐risk pregnancies in experienced NIPT centers.

Both MiSeq and NextSeq platforms are suitable for Clarigo test. Majority of our cases are investigated by MiSeq platform. But, the NextSeq workflow is cheaper and definitely easier in wet lab. Also “uncertain results,” “rate of manual reports,” and “test failure rates” are less in contrast to MiSeq platform. In addition, NextSeq has clear advantage of high sample coverage which is necessary for reliable reporting (Table 3). MiSeq may be preferred by centers with low number of cases.

Test failure rates could be minimized by repetition of the test; half of the failed tests are saved in second repeat. But we restricted the repetition number by two because of the limited government funding. LFF is the main reason (50%) for failure and waiting for increase in fetal fraction and resampling is not preferred generally. Because, the fetal fraction increases slightly (0.1% per week) with gestational age until 20 weeks, and then increases just 1% per week (Wang et al., 2013). But in practice, wrong sampling is common in unexperienced centers, and the resampling has a rationale in cases with inappropriate cfDNA concentrations. The preservatives in Streck tubes stabilize the cell membranes, keep maternal nuclear genomic DNA inside the cells, and avoid dilution of fetal cfDNA in the plasma. So, mixing the blood gently with preservatives inside the tube is the initial and a major step in the laboratory workflow. In our study, LFF is responsible for the test failure in 23% of MiSeq group and 26% in NextSeq group. In total, “Low cfDNA concentration” and “LFF” constitute 42.5% (204/480) of the failure factors. Concerning other failure reasons, “Uncertain Result for Trisomy 21” is the main obstacle for MiSeq and “Rejection Due to Low Sample Correlation” is for NextSeq. For our country, high prevalence of parental consanguinity, obesity (Wang et al., 2013), and liberal prescription of low‐molecular weight heparin (Burns et al., 2017) may also undermine the NIPT.

Uncertain result for trisomy 21 or other failed results necessitate invasive investigation which is 8.7% in NextSeq group. The ratio is more than literature (1%–5%). The increased failure rate could be related with limitation of test repeats due to restricted funding or inexperienced center. In pregnants with moderate risk, failure rate is an important issue of pretest counseling. For an uninformed couple it is easy to prefer a noninvasive test, but unexpectedly the failed test may lead to an invasive procedure.

Generally medical authorities are against nonmedical use of prenatal testing to accommodate parental curiosity about fetal sex (Amant et al., 2015). Our legal regulations also prohibit “fetal sex determination” and we do not share the fetal sexes in our reports except for very rare medical exceptions.

Fetal cfDNA screening is the most sensitive screening method for common trisomies, more than 99% trisomy 21 detection rates are achieved by distinct methods (Burns et al., 2017). As far as we know, we have two false negatives in 4,594 pregnancies: One fetus with trisomy 18 which is diagnosed by fetal ultrasonography and subsequent invasive test; one fetus with trisomy 21 which is diagnosed by physical examination of neonate. In addition, NIPT decreases the number of invasive procedures dramatically. The number of amniocentesis decreased 53% in our hospital (1,500–800/year). Targeted NIPT approach differs by its cost which is necessary if restricted financial sources are present. Also, the huge number of cases may be concluded in a single‐targeted NGS run. The finite number of targeted SNPs (4,000) makes it possible to analyze 192 cases per high‐throughput NextSeq run in 3 days. The feature is essential for a screening test. As a conclusion, our experience with an in house test reveals that targeted NIPT by NGS platforms with high capacity is an effective option for screening of common trisomies. The future solutions may also be used to detect CNV. But in our point of view, CNVs seem to be the concern of prenatal microarrays. The NIPT should focus on trisomy 21, by this way, they could be cheaper and the failure rates may be decreased.

## CONFLICT OF INTEREST

None declared.
