# Supplementary material for: Targeted fetal cell‐free DNA screening for aneuploidies in 4,594 pregnancies: Single center study
Source: Mol Genet Genomic Med. 2019 May 8;7(7):e00678. doi: 10.1002/mgg3.678 (PMC6625369; doi:10.1002/mgg3.678)
Supplement: Supplementary file 1 [file MGG3-7-e00678-s001.doc]

Genetik bozuklukların ve doğuştan özürlerin taranması tüm gebelere önerilmektedir. Ultrason gibi tarama yöntemleri rutin olarak uygulanmaktadır. Down sendromu taramasında kullanılan kan testleri gibi bazı tarama testlerinin yapılması ise hasta tercihine göre yapılır. Tarama yapılmasına ilişkin verilecek kararlar oldukça kişiseldir, önem verilen değerlere göre ve kişisel beklentilere göre değişir. Duygu ve düşüncelerinizi kadın doğum uzmanı ile görüşmeniz size yardımcı olabilir. Sonuçlarından nasıl faydalanacağınızı da konuşmak isteyebilirsiniz. Bu konuşmaya rehberlik etmesi için bazı üzerinde düşünülmesi gereken soruları ve cevapları ana hatları ile bilginize sunduk.

**Genetik bozuklukların araştırılması için doğum öncesi tarama yaptırmalı mıyım?**

Beklentilerinize ve önem verdiğiniz değerlere göre tarama testlerinin yapılmasını veya yapılmamasını tercih edebilirsiniz. Doğum öncesi taramanın bir faydası sağlık sorunları olan veya özrü olan bir çocuğu yetiştirmeye hazırlanmak olabilir. Bir başka faydası rahatsızlıkla ilgili bilgi edinmek veya doğum planları yapmak olabilir. Bazı aileler, doğum sonrası erken tedaviye başlamak isteyebilir veya bazen aileler yaşamayacak bir çocuğa kendilerini hazırlamak isteyebilirler. Bazı aileler gebeliğin sonlandırılmasını veya özürlü çocuğun evlatlık verilmesini tercih edebilirler. Kimi aileler hiçbir doğum öncesi tarama testinin yapılmamasını isteyebilir. Bu aileler testlerin işe yaramadığını veya fazlaca strese ve endişeye sebep olduğunu düşünebilirler.

**Serbest DNA Taraması (SDT) nedir?**

Bazı genetik hastalıkların araştırılması için serbest DNA taraması bir seçenek olarak size önerilebilir. Serbest DNA taraması “Girişimsel olmayan doğum öncesi test/tarama (NIPT/S)” olarak da adlandırılmaktadır. Bazen bu teste özel ticari isimler de verilebilmektedir. Serbest DNA taraması gebelerden kan örneği alınarak yapılmaktadır ve düşük riski taşımamaktadır. Bu kan testinin sonucu çoğunlukla doğru olmakla birlikte, elde edilen sonuç kesin değildir. Serbest DNA taraması, bebeğiniz genetik hastalık taşısa bile normal sonuç verebilir (yanlış negatif). Diğer yandan, test bir hastalık için yanlış şekilde yüksek risk bildirebilir (yanlış pozitif). Bu nedenle kesin sonuç almak isteyenlere tanı testi önerilmektedir. Ayrıca, serbest DNA taraması, tüm genetik hastalıkları veya bir gebelikte gerçekleşebilecek tüm olası riskli durumları göstermez. Sonuçlar bu konuda uzman olan bir tıp hekimi ile birlikte değerlendirilmelidir.

Down Sendromu için kullanılan ikili, üçlü, dörtlü test gibi pek çok farklı kan testi size önerilebilir. Aynı anda birden fazla tarama testi yaptırmamalısınız. Kadın doğum uzmanınız sizinle her bir seçeneğin yararlarını ve sakıncalarını tartışacaktır. Bu testler ile ilgili sorularınız için kadın doğum uzmanınıza başvurun.

**Tanı testi nedir? Tarama testinden ne farkı vardır?**

Tanı testleri, gerçeğe en yakın sonucu veren testlerdir. En doğru sonuca ulaşmak isteyen tüm gebeler tanı testi yaptırabilir. Tanı testlerinin doğruluk oranı %99’un üzerindedir ve SDT sonuçlarının sağlaması için kullanılabilir. Bu testler tarama testlerinde tespit edilemeyecek genetik durumları gösterebilir. Gebelik haftasına göre 3 tanı testi seçeneği bulunmaktadır: Koryonik Villus Örneklemesi, Amniyosentez, Kordosentez. Bu testler girişimsel testlerdir ve bu testlerle bebeğinizin kromozomlarını gösterebilmek için az bir miktar plasenta dokusuna, amniyon sıvısına veya göbek bağından alınacak kana ihtiyaç vardır. Tanı testleri girişimsel oldukları için %1 civarında düşük tehlikesine sahiptir.

**Serbest DNA Taraması ile hangi durumlar araştırılmaktadır?**

Serbest DNA taramasının esas hedefi Down Sendromu (Trizomi 21), Trizomi 18 (Edward Sendromu) ve Trizomi 13 (Patau Sendromu) taramasıdır. Fetusun cinsiyetinin belirlenmesi ülkemizde yasaktır. Cinsiyetle ilişkili hastalıklarda istisnai olarak cinsiyet belirlemesi yapılabilir. Serbest DNA taraması kesin sonuç vermemekle birlikte, en etkin Down Sendromu taraması için kullanılmaktadır. Ancak SDT’nin yukarıda bahsedilen hastalıklar dışında da kullanılabilmesi için çalışmalar devam etmektedir. Hekiminizden bu konuda bilgi alabilirsiniz.

Kromozom bozukluğu olan bebeklerde çeşitli ciddi hastalıklar görülmektedir. Örneğin, trizomi 13 ve 18 hastalarının ağır zeka ve sağlık sorunları olmaktadır; yalnızca %10’u bir yıldan uzun yaşayabilir. Down Sendromlular genelde hafif-orta şiddette gelişme geriliğine sahiptir ve hastaların bir kısmının tedavi edilebilir sağlık sorunları vardır; az bir kısmında ise daha ciddi sorunlar vardır. Genelde toplumda aktif olarak yer alırlar ve ortalama 60 yıl yaşarlar. Cinsiyet kromozomlarında fazlalık veya eksiklik olması bazen öğrenme güçlüklerine ve sağlık sorunlarına sebep olabilir; fakat çok hafif seyreden ve teşhis edilmeden kalan bozukluklar da vardır.

**Serbest DNA Taramasının sonuçları ne kadar zamanda çıkar?**

Genel olarak 15 gün içinde sonuç alınır. Genetik veya kadın doğum uzmanınızdan sonuç raporunun ne zaman çıkacağı ve raporunuzu nasıl alacağınızı öğrenebilirsiniz.

**Serbest DNA Taramasının sonuçları ne anlama gelir?**

“Negatif” sonuç, bebeğinizin araştırılan genetik hastalıklar için düşük risk taşıdığı anlamına gelir. Riskin olmadığı anlamına gelmez; SDT tüm hastalıkları taramaz. “Pozitif” SDT sonucu, bebeğinizin bir hastalığa sahip olma ihtimalinin yüksek olduğunu bildirir. Pozitif bir sonuç sonrası hastalıkla karşılaşma riskiniz, hastalığa, yaşınıza, gebelik haftanıza, aile hikayenize ve ultrason sonuçlarınıza göre değişir. Bazen SDT testi sonuç vermeyebilir. Sonuç alamadığınızda bunun ne ifade ettiğini genetik veya kadın doğum uzmanınıza sorabilirsiniz.

**Test sonucum, bir genetik hastalık için “pozitif” veya “yüksek riskli” çıkarsa nasıl bilgi ve destek alabilirim?**

Doğum öncesi tarama testleri gebeliğin seyri hakkında size bilgi verirken, aklınıza pek çok soru gelmesine de sebep olabilir. Örneğin: Bu sonuçlar ne anlama gelmektedir? Bu hastalığa sahip bir insanın yaşamı nasıl olur? Güvenilir bilgiyi nereden alabilirim?

Hastalıklar hakkında araştırma yaparken yanlış veya güncel olmayan bilgilere ulaşabilirsiniz. Son yıllarda genetik hastalıklarla ilgili büyük gelişmeler olmuştur. Ailelerin güncel bilgiye ulaşabilmesi için bir kadın doğum uzmanından bilgi alması ve onun gösterdiği güvenilir kaynaklardan bilgi edinmesi uygundur.

Daha detaylı bilgi almak için genetik danışmanlık almak isteyebilirsiniz. Genetik danışmanlığı, doğum öncesi genetik hastalıklar konusunda bilgili genetik uzmanı tıp hekimleri vermektedir. Genetik uzmanları test seçenekleriniz hakkında ve sizin test ile ilgili düşünceleriniz hakkında sizinle görüşebilirler. Test sonuçlarınız hakkında doğru bilgi almanızı sağlarlar. Genetik uzmanlarına kadın doğum uzmanınızın yönlendirmesi ile veya http://www.tibbigenetik.org.tr/ sitesinden ulaşabilirsiniz.

Hastanemizde serbest DNA taraması testinin kimlere, ne zaman yapılabileceği genetik hastalıklar ve perinatoloji (riskli gebelik) polikliniklerince belirlenmektedir.

Bu doküman “Ulusal Genetik Danışmanlar Topluluğunun” (National Society of Genetic Counselors, http://nsgc.org/) hastalar için oluşturduğu bilgilendirme rehberi esas alınarak hazırlanmıştır.
