# Supplementary material for: Targeted fetal cell‐free DNA screening for aneuploidies in 4,594 pregnancies: Single center study
Source: Mol Genet Genomic Med. 2019 May 8;7(7):e00678. doi: 10.1002/mgg3.678 (PMC6625369; doi:10.1002/mgg3.678)
Supplement: Supplementary file 2 [file MGG3-7-e00678-s002.doc]

**İSTEM TARİHİ: ……../……../…………….**

| **Hastanın Adı Soyadı:**  **TC No:** | **Ağırlığı (kg), Boyu (m):**  **Vücut Kitle İndeksi (kg/m^2^):** |
| --- | --- |
| **Son Adet Tarihi:**  **Gebelik Haftası (USG):** | ***Endikasyonlar***  **Yaşı:**  **NT (mm):**  **İkili Test Riski:**  **Üçlü Test Riski:**  **Dörtlü Test Riski:** |
| **Ek Klinik Notlar:**  **Kaşe ve İmza** | |
| **Uyarılar: Fetal USG anomalisi olan veya NT>3,5mm** olan gebeliklerde tarama yöntemleri yerine girişimsel (amniyosentez vb.) tanı testlerinin yapılması önerilmektedir.   - Çoğul gebeliklerde (ikiz, vanishing twin vb.), - Oosit donasyonu ile oluşan gebeliklerde, - Gebelik haftası 8 haftanın altında olduğunda, - Vücut kitle indeksi ≥ 35 olan veya - Kromozom anomalisi olan anne adaylarında, - Son 3 ay içerisinde, kan nakli yapılan, transplantasyon yapılan, kök hücre tedavisi gören, immunoterapi veya radyoterapi alan gebelerde **“Serbest DNA Taraması” önerilmemelidir.**   Maternal malinitede yanlış pozitiflikler görülebilir.  Test fetal cinsiyet kromozomu bozukluklarını göstermez.  Fetal fraksiyonun %4’ün altında olduğu durumlarda test sonuç vermez. | |

***Formu Eksiksiz Doldurunuz. Dahili Telefonlar: Sekreterlik 4035, Poliklinik 4006.***
