# Supplementary material for: Targeted fetal cell‐free DNA screening for aneuploidies in 4,594 pregnancies: Single center study
Source: Mol Genet Genomic Med. 2019 May 8;7(7):e00678. doi: 10.1002/mgg3.678 (PMC6625369; doi:10.1002/mgg3.678)
Supplement: Supplementary file 3 [file MGG3-7-e00678-s003.docx]

**İzmir Tepecik Eğitim ve Araştırma Hastanesi Genetik Hastalıklar Tanı Merkezi**

**Serbest DNA Taraması (NIPT) Raporu**

| **Genetik Dosya No** |  | **Barkod** |  |
| --- | --- | --- | --- |
| **Adı soyadı** |  | **Materyal Alış Tarihi** |  |
| **Doğum Tarihi** |  | **Materyal Kabul Tarihi** |  |
| **TC No** |  | **Kabul Notu** |  |
| **Protokol No** |  | **SUT Kodu** |  |
| **Endikasyonu** |  | **Rapor No** |  |
| **Gebelik Haftası** |  | **Rapor Tarihi** |  |

**Yöntem:** Çalışma anne kanından, plazmada bulunan serbest DNA fragmanları kullanılarak, fetal anöploidi taraması için yapılmıştır. Test hedefe yönelik (targeted) tasarıma sahip Clarigo Test (CE-IVD) ile masif paralel sekans sistemleri kullanılarak ve örnek başına yaklaşık 2M okuma yapılarak tamamlanmıştır. Elde edilen sonuçların raporlanmasında Clarigo Reporter yazılımının algoritmaları kullanılmıştır.

**Sonuçlar:**

| Trizomi 21 (Down Sendromu) | Negatif/Belirsiz/Pozitif |
| --- | --- |
| Trizomi 18 (Edward Sendromu) | Negatif/Belirsiz/Pozitif |
| Trizomi 13 (Patau Sendromu) | Negatif/Belirsiz/Pozitif |
| Fetal Fraksiyon (%) | 0 |

**Yorum:**

Test tüm doğuştan hastalıkları taramaz. Kişiye özel NPV ve PPV değerleri. “Negatif” sonuç, bebeğinizin araştırılan genetik hastalıklar için düşük risk taşıdığı anlamına gelir; riskin olmadığı anlamına gelmez. “Pozitif” sonuç, bebeğinizin bir hastalığa sahip olma ihtimalinin yüksek olduğunu bildirir ve amniyosentez gibi bir girişimsel tanı testi ile bu sonucun doğrulanması gerekir. Test sonucu çıkmayan (belirsiz) gebelerin de fetal anöploidi riski yüksek olması nedeniyle, ayrıntılı fetal USG ve girişimsel tanı testi yaptırmaları önerilir. Anneye ait translokasyon ve mikrodelesyonlar test sonuçlarının yanlış çıkmasına sebep olabilir. Fetusa ait mozaik genetik değişiklikler veya parsiyel kromozom anöploidileri “yanlış negatif” sonuçlara sebep olabilir. Plasental mozaisizm, kaybolan ikiz (vanishing twin) ve maternal malinite gibi durumlarda “yanlış pozitiflikler” görülebilir.
